# Intelligent Soft Sensors

**DOI:** 10.3390/s23156895

**Published:** 2023-08-03

**Authors:** Simon Tomažič

**Affiliations:** Faculty of Electrical Engineering, University of Ljubljana, 1000 Ljubljana, Slovenia; simon.tomazic@fe.uni-lj.si; Tel.:+386-1-4768-760

In this Special Issue, we embark on a journey into the exciting field of intelligent soft sensors, and take a deep dive into the groundbreaking advances and potential that these software algorithms have introduced in various fields. Soft sensors, often referred to as virtual sensors, are fast becoming a cornerstone of the ever-evolving digital age. To appreciate the capabilities of soft sensors, one must acknowledge their fundamental basis. These tools are based on sophisticated mathematical models, state-of-the-art computational techniques, and rigorous algorithmic strategies. Fields as diverse as physics, electrical engineering, biology, mathematics and even economics have influenced their development, making them a true example of interdisciplinary excellence.

Because these algorithms process a large number of measurements simultaneously, insights into online estimation of complex process variables that were previously impossible have become possible. In areas such as control applications, fault detection or data fusion, the importance of soft sensors has increased. Essentially, data from numerous sources are fused to gain insights from the apparent clutter.

The broader socio-economic and environmental context must also be considered. With rising costs, increasing concerns about sustainability and the overarching need for efficiency, the importance of soft sensors is clearer than ever. They provide the adaptability necessary for optimised resources, minimised waste and improved performance, meeting the pressing needs of the 21st century. In the rapidly digitising world, the ingenuity and breakthrough capabilities of today’s researchers and engineers are undeniably impressive. It is not just about finding solutions to current problems, but also about identifying opportunities that many have not yet recognized. The forward-looking nature of this field becomes even clearer in the context of soft sensors. With this technological development, it is important to also recognise the broader impacts and applications.

Among the many interesting contributions in this Special Issue, one deals with the complexity of setting observer parameters for second-order systems with constant perturbations [1]. A novel algorithm that simplifies the inherent complexity in defining the convergence rate and the range for unknown state errors is the focus of the discussion. By matching the theory with practical scenarios, its usefulness is highlighted using a microalgae bioreactor model, emphasising the effectiveness of the algorithm in ensuring an optimal convergence rate and precision. The challenges posed by unobservable and nonlinear systems are addressed in [2], which presents a unique extended Kalman filter framework. An intelligent projection onto an observable subspace is used to solve previously insurmountable instability problems. The brilliance is particularly evident in sensor selection, which helps stabilise unobservable states and optimise sensor selection without the need for actual measurements. A deeper understanding is fostered by another valuable contribution [3] that re-evaluates conventional prediction and health management techniques. In an era characterised by rapid production cycles and frequent design changes, the urgency of a more dynamic, self-contained test optimisation methodology is highlighted. By merging soft sensing, ensemble learning and extreme learning machines (ELMs), a technique is presented that excels in efficiency and adaptability to the intricacies of modern systems.

However, the applications of soft sensors are not limited to manufacturing or engineering. Their impact on the medical and psychological fields is equally significant. The research [4] presented on ToLA—a computerised adaptive testing version of the Tower of London task—is exemplary. An item response theory was combined with an adaptive algorithm. The result is a diagnostic tool with increased sensitivity that provides a deeper understanding of neurodevelopmental disorders. Similarly, a model for measuring hypnotic depth during intravenous general anaesthesia is presented [5], pointing to a future where anaesthetic treatments are perfectly individualised.

Research into electrodermal activity (EDA) for stress detection [6] highlights the importance of monitoring mental health. The application of frequency spectrum analysis in this research promises a fast, accurate and efficient assessment of stress in real time, which is particularly important given the global increase in mental health problems.

In bioprocessing, where the goal is to improve accuracy and efficiency, soft sensors are becoming increasingly useful, especially when combined with Raman spectroscopy and machine learning. A study on the automated feeding of Chinese hamster ovary cells (CHO) [7] is presented, highlighting the importance of integrating Raman spectroscopy and chemometrics to develop models for monitoring and controlling CHO bioprocesses. The profound implications of the careful preprocessing and wise selection of multivariate analysis methods to improve accuracy in the monitoring of important process variables are demonstrated.

To further explore the topic of bioprocesses, the fermentation process of Pichia pastoris is examined and the BDA-IPSO-LSSVM soft sensor modelling method is presented [8]. This model addresses the recurring problem of model errors due to discrepancies in data distribution. By leveraging the combined strengths of transfer learning, fuzzy set concepts and advanced optimisation techniques, exceptional predictive accuracy is achieved, even under changing conditions. While these advances are remarkable, the transformative impact of soft sensors extends even further in the modern technology landscape.

With the increasing integration of technologies such as artificial intelligence, machine learning and IoT (Internet of Things), the potential of soft sensors is growing exponentially. Possibilities that were once considered unattainable are now becoming tangible. It is not just about the sensors, but also about the ecosystems that enable them. Change is on the horizon as the industry is ready to adopt a proactive maintenance culture, predictive quality control and instant adaptive adjustments. In the field of soft sensor research, societal impact and directions are becoming increasingly important. Educational institutions, companies and policy makers play a crucial role in shaping this path. Their joint efforts to create an enabling environment foster innovation and drive progress in this dynamic field.

The following section looks at the versatility and scope of soft sensor applications in industry. The research on self-acting shape memory coils (SMC) with variable stiffness [9] is worth highlighting. The proposed method not only improves the performance and cost efficiency of SMA actuators, but its applicability extends across various fields from robotic grippers to biomedical devices.

The next paper [10] deals with the merging of data from different sources in sintering processes and highlights the growing importance of integrating different types of data for better predictions. By cleverly merging visual data with traditional time series process data, the method achieves significant improvements in predicting complicated quality variables such as FeO content. In the area of safety and infrastructure, research on a highly sensitive fire detection and early-warning system [11] highlights the value of fusing multiple fire indicators for timely and effective fire detection in buildings. This hybrid feature-fusion-based strategy represents potential for much safer smart building systems.

The essence of soft sensors lies in the data they interpret. These are not just any data, but a mixture of signals, inputs and variables that all help to describe the underlying processes. When these nuances are unravelled, soft sensors reveal the hidden subtleties of the world. Whether it is the microenvironment of a bioreactor, the status of a complicated machine or the subtle changes in human physiology, these virtual tools enable a comprehensive understanding.

In addition, much attention is being paid to ethics and transparency in relation to these technologies. As these sensors play an increasingly central role in determining outcomes, such as autonomous driving decisions—whether to avoid an obstacle or brake abruptly—the need for transparency, accountability and ethical considerations in their algorithms becomes ever more important. It is our collective responsibility to ensure that the full potential of these technologies is realised while adhering to ethical principles and practices.

In summary, the articles in this Special Issue not only highlight the advancing frontiers of soft sensors in various fields, but also emphasise the importance of multidisciplinary collaboration in these developments. As technologies advance, so will the applications and opportunities for soft sensors. Challenges and opportunities await researchers and professionals in this field, as the future promises even more advanced and integrated solutions.

## Data Availability

Not applicable.
